# Tooth Culture

**Published:** 1892-09

**Authors:** 


### Tooth Culture.

In a capital address on "Tooth Culture," delivered at the annual meeting of the Eastern Counties Branch of the British Dental Association, printed in the current number of the *Lancet*, Sir James Crichton-Browne referred to a change which has taken place in bread, as one of the causes of the increase of dental caries. So far as our own country is concerned, this is essentially an age of white bread and fine flour, and it is an age therefore in which we are no longer partaking, to anything like the same amount that our ancestors did, of the bran or husky parts of wheat, and so are deprived to a large degree of a chemical element which they contain—namely, fluorine. The late Dr. George Wilson showed that fluorine is more widely distributed in nature than was before his time supposed ; but still, as he pointed out, it is but sparingly present where it does occur, and the only channels by which it can apparently find its way into the animal economy are through the siliceous stems of grasses and the outer husks of grain, in which it exists in comparative abundance. Analysis has proved that the enamel of the teeth contains more fluorine, in the form of fluoride of calcium, than any other part of the body, and fluorine might, indeed, be regarded as the characteristic chemical constituent of this structure, the hardest of all animal tissue, and containing 95.5 per cent. of salts, against 72 per cent. in the dentine. As this is so, it is clear that a supply of fluorine, while the development of the teeth is proceeding, is essential to the proper formation of the enamel, and that any deficiency in this respect must result in thin and inferior enamel. Sir James Crichton-Browne thinks it well worthy of consideration whether the reintroduction into our diet of a supply of fluorine in some suitable natural form—and what form, he asks, can be more suitable than that in which it exists in the pellicles of our grain stuffs?--might not do something to fortify the teeth of the next generation.

